# Multilevel examination of minor salivary gland biopsy for Sjögren's syndrome significantly improves diagnostic performance of AECG classification criteria

**DOI:** 10.1186/ar1486

**Published:** 2005-01-17

**Authors:** Patrizia Morbini, Antonio Manzo, Roberto Caporali, Oscar Epis, Chiara Villa, Carmine Tinelli, Enrico Solcia, Carlomaurizio Montecucco

**Affiliations:** 1 Department of Pathology, IRCCS Policlinico S Matteo, Pavia, Italy; 2 Department of Rheumatology, IRCCS Policlinico S Matteo, Pavia, Italy; 3 Biometric Unit, IRCCS Policlinico S Matteo, Pavia, Italy

**Keywords:** focus score, minor salivary gland biopsy, multilevel examination, Sjögren's syndrome

## Abstract

The recently observed low reproducibility of focus score (FS) assessment at different section depths in a series of single minor salivary gland biopsies highlighted the need for a standardized protocol of extensive histopathological examination of such biopsies in Sjögren's syndrome. For this purpose, a cumulative focus score (cFS) was evaluated on three slides cut at 200-μm intervals from each of a series of 120 salivary biopsies. The cFS was substituted for the baseline FS in the American–European Consensus Group (AECG) criteria set for Sjögren's syndrome classification, and then test specificity and sensitivity were assessed against clinical patient re-evaluation. Test performances of the AECG classification with the original FS and the score obtained after multilevel examination were statistically compared using receiver operating characteristic (ROC) curve analysis. The diagnostic performance of AECG classification significantly improved when the cFS was entered in the AECG classification; the improvement was mostly due to increased specificity in biopsies with a baseline FS ≥ 1 but <2. The assessment of a cFS obtained at three different section levels on minor salivary gland biopsies can be useful especially in biopsies with baseline FSs between 1 and 2.

## Introduction

Sjögren's syndrome (SS) is characterized by diffuse chronic inflammation of exocrine glands, which leads to symptoms and complaints referred to as 'sicca syndrome' [1]. No single instrumental or laboratory parameter is available for the diagnosis of SS, which relies instead on the evaluation of multiple clinical, serological, functional, and morphological parameters [2], such as those proposed and validated by a group of investigators sponsored by the European Community (now the European Union) [3,4] and recently revised by the American-European Consensus Group (AECG) [5]. The presence of chronic inflammatory infiltrates in lip salivary glands, as assessed with minor salivary gland biopsy (MSGB), is one of the parameters included in most criteria sets proposed for SS classification [3,5-9]. Salivary gland inflammation is assessed by scoring the degree of infiltration according to the method of Greenspan and Daniels, who defined the focus score (FS) as the number of inflammatory infiltrates of at least 50 cells present in 4 mm^2 ^of gland surface unit [10,11]. Different criteria sets consider as positive a FS ≥ 1 or FS ≥ 2 [3,9]. Although the methodology of sampling, processing, and examining MSGBs has been standardized [10,11], the reproducibility of the routine histopathological evaluation in the diagnosis of SS at different section levels within the same biopsy specimen has been recently challenged [12,13]. To avoid any bias that might therefore arise, the examination of multiple levels of tissue has been recommended, to maximize the number of foci, the glandular area, and the technical quality of the material, although the number of sections required has not yet been standardized [12].

In this study, we tried to standardize a protocol for histopathological MSGB evaluation in which the FS is assessed by examining a larger area of the biopsy tissue, and we investigated how the FS obtained affects the number of patients classified as having SS, as compared with the routine method, using the classification criteria recently proposed by the AECG [5]. The diagnostic accuracy of the test was validated against the clinical re-evaluation of the patients performed by two experienced rheumatologists after at least 1 year of follow-up.

## Materials and methods

### Selection criteria

We retrospectively studied a consecutive series of patients thoroughly investigated at our hospital between 1998 and 2002 for suspected primary SS, including a follow-up of at least 1 year after the diagnostic evaluation. Patients with secondary SS or who had been diagnosed by biopsy as having nonspecific inflammation, fibrosis, and atrophy of the gland were excluded [10-12]. Less-than-optimal tissue area (biopsy section area less than 4 mm^2^) was not considered a criterion for exclusion, provided that at least one normotrophic glandular lobule had been sampled.

### Baseline clinical and histopathological evaluation

All patients had undergone thorough clinical and instrumental evaluation [3,4], including MSGB performed as suggested by Daniels [11]. The diagnosis of SS was established for all patients according to the classification criteria proposed by the AECG [5]. MSBG samples were fixed in formalin, processed, and embedded in paraffin according to standardized laboratory methods. Baseline histopathological slides containing 4-μm-thick sections stained with hematoxylin and eosin were reviewed by a pathologist, blinded to clinical and laboratory data, who recorded for each patient the number of glands, the sample surface area, the presence of alterations suggestive of nonspecific sialoadenitis, and the baseline FS [10,11]. The lymphocytic focus and the focus score were defined according to Greenspan and Daniels [10,11]. In individual biopsies, lobules with acinar atrophy and diffuse fibrosis were excluded from diagnostic evaluation. The histological parameter was considered as negative in the absence of any inflammatory infiltrate (FS = 0) and in the presence of less than 1 focus per 4 mm^2 ^(0 < FS < 1) [5]; the presence of one or more foci per 4 mm^2 ^was considered positive when the adjacent glandular parenchyma was histologically normal. We further classified patients with a positive FS into two groups, those with fewer than two foci per 4 mm^2 ^(1 ≤ FS < 2) and those with two or more (FS ≥ 2). The area of the biopsy sections was assessed with video-assisted morphometric software capable of measuring the area of delineated surfaces (ImageDB System, Casti Imaging, Cazzago di Pianiga, Italy). The comparison of automated and manual area measurements of a smaller series of MSGB sections did not show a significant difference (data not shown). This prompted us to choose the automated system to simplify the examination of the large number of samples involved in the study.

### Serial histopathological re-evaluation

Sample blocks were recut at two additional levels, about 200 and 400 μm deeper than the original section. Sections 4 μm thick corresponding to these levels were collected on separate slides and stained with hematoxylin and eosin. Considering that an infiltrate of 50 lymphocytes in our section had a mean diameter of 50 μm, we assumed that the interposition of 200 μm between the evaluated sections was enough to ensure that the FS recorded at each level was independent of the other two and that if the same focus was present in two section levels, the focus itself was large enough to justify repeated scoring. The two new sections were blindly examined by the same pathologist, who again recorded the area and the focus score for each level. For each patient, the total number of foci at all three levels and the total surface area measured at all levels were used to calculate a cumulative FS (cFS) for the three sections.

### Reclassification of patients

The cFS obtained after re-evaluation was entered in the AECG criteria set [5], to obtain a re-classification of each patient. To compare the diagnostic performance of the original classification and the reclassification, a 'gold standard' was needed independent of the AECG criteria set. We adopted as reference standard the opinion of experienced clinicians, analogously to what had been done by the European Community Study Group on Diagnostic Criteria for Sjögren's Syndrome when SS and control patients were selected to validate the proposed criteria [3-5]. Briefly, three experienced rheumatologists, blinded to the results of the histopathological re-evaluation, performed a clinical evaluation of each patient and reviewed the patient's charts including the original clinical, laboratory, and instrumental evaluation, and the subsequent documentation covering at least 1 year of follow-up and treatment response. On this basis they were requested to judge whether individual patients had SS.

### Statistical analysis

Quantitative data are shown as means ± standard deviation (SD). Specificity and sensitivity were assessed with their 95% confidence intervals (CI). Differences in frequencies were evaluated by means of chi-square statistics or the Fisher exact test, as appropriate. Given the known limitations of diagnostic accuracy as a parameter for measuring the diagnostic performance of a test, specificity and sensitivity were compared using receiver operating characteristic (ROC) curves [14]. A *P *value of less than 0.05 was considered to indicate statistical significance. All tests were two-sided. Analyses were performed with Statistica for Windows (StatSoft Inc, 2002, Tulsa, OK, USA) and MedCalc software.

## Results

### Baseline examination

The study series comprised 138 patients, 65 of whom had a baseline FS = 0, 14 with 0 < FS < 1, 18 with 1 ≤ FS < 2,  and 41 with FS ≥ 2. Eighteen patients had incomplete clinical data that hampered either the AECG classification or the clinical re-evaluation. These patients (8 with FS = 0, 3 with 0 < FS < 1, 3 with 1 ≤  FS < 2, and 4 with FS ≥ 2) were excluded from further analysis. The final series included 120 patients, for whom demographic, biopsy, and clinical data and the result of the clinical re-evaluation are presented in Table 1.

### Histological re-evaluation

In 96 (80%) of the 120 biopsies, the FS group did not change after serial sectioning and calculation of the cFS. In 14 of these biopsies, the FS group changed but this did not affect that patient's negative or positive status. In the biopsies for the other 10 patients, 1 (1.7%) of the 57 with a baseline FS = 0 and 1 (9%) of the 11 with a baseline score of 0 < FS < 1 switched to a FS consistent with SS according to AECG criteria (FS ≥ 1). At clinical re-evaluation, these two patients were considered not to have SS. Seven (46%) of the 15 patients with a baseline score of 1 ≤  FS < 2 and one (3%) of 37 with a baseline FS ≥ 2 switched to a grade inconsistent with SS (FS < 1). On clinical re-evaluation, 7 of these 8 patients were assessed as not having SS.

### Patient reclassification according to AECG criteria

When the cFSs were entered in the AECG criteria set [5], the baseline classifications of the 63 non-SS patients were not changed, while the classifications of 7 of the 57 patients originally classified as having SS were changed to non-SS (Table 2). The classification was changed in 6% of the 120 patients. Six of these seven patients had a baseline score of 1 ≤ FS < 2 and one had a baseline FS ≥ 2. On clinical re-evaluation, all these seven patients were judged not to have SS. The clinical re-evaluation also refuted 7 of the 113 (6.2%) classifications that had not been changed at biopsy revision. Considering the clinical re-evaluation as the reference gold standard, the number of false-negative AECG classifications did not change (3 of 63 AECG non-SS cases), while the number of false positives was reduced from 11 to 4 (63.6% reduction).

### Comparison of sensitivity and specificity between baseline and multilevel FS evaluation

**Figure 1:**
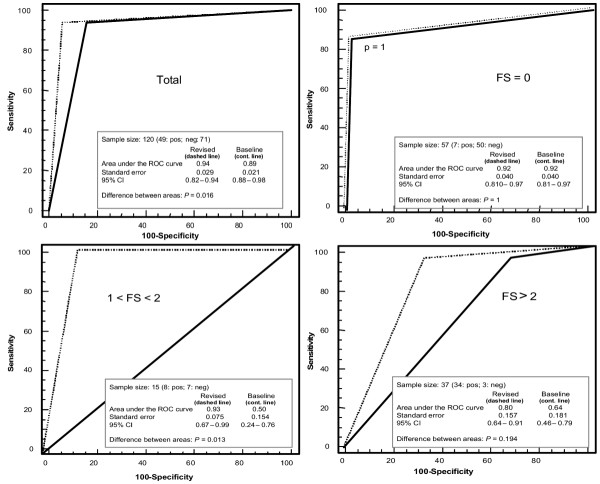
Statistical comparison of the diagnostic performance of the American-European Consensus Group (AECG) criteria for Sjögren's syndrome with baseline and cumulative focus scores (FSs). Receiver operating characteristic (ROC) curves were used to compare the sensitivity and specificity of the AECG criteria with the baseline focus score and with the FS obtained after multilevel histopathological evaluation, with respect to the gold standard of patient re-evaluation by the experienced rheumatologists. The diagnostic performance was significantly improved in the overall series (top left panel; *P= *0.016), mostly because of the improvement in the group of patients with 1 ≤ FS < 2 (bottom left; *P= *0.013). No difference was observed when FS = 0. No ROC curve could be obtained in the group of patients with 0 < FS < 1, because of the absence of cases classified as Sjögren's syndrome at clinical re-evaluation (positive gold standard). CI, confidence interval.

In the present series of 120 patients fully evaluated for SS, the sensitivity and specificity of the baseline AECG criteria set were 93.9% and 84.5%, respectively. Reclassification with cFS did not affect sensitivity, whereas specificity changed to 94.4% (*P = *0.056), increasing the accuracy from 88.3% (95% CI 81.2–93.5) to 94.2% (95% CI 88.3–97.6). Pairwise comparison of the ROC curves showed a statistically significant difference between patient classification before and after multilevel FS evaluation (difference between areas: 0.049 [SE 0.021]; 95% CI 0.009–0.089; *P = *0.016) (Fig. 1). Sensitivity and specificity did not change for biopsies with FS = 0 or FS < 1 (inconsistent with SS), while specificity increased substantially in biopsies consistent with SS (FS ≥ 1) (Table 2). Pairwise comparison of the ROC curves showed a statistically significant difference (*P = *0.013) only in biopsies with 1 ≤ FS < 2 (difference between areas: 0.43 [SE: 0.17]; 95% CI 0.09–0.76; *P = *0.013; Fig. 1). The diagnostic accuracy of the MSGB histological analysis considered independently of other criteria changed from 85.8% (95% CI 78.3–91.5) to 90.8% (95% CI 84.2–95.3), but the comparison of the ROC curves did not show a statistically significant difference (*P = *0.15).

## Discussion

In the present study, we show that the histopathological evaluation of salivary gland biopsies with multilevel sectioning and assessment of a cumulative focus score (cFS) changes the baseline classification in 6% of patients evaluated for SS and increases the diagnostic performance of the criteria recently proposed by the AECG for SS classification [5]. In particular, multilevel evaluation improved the diagnostic accuracy of biopsies with a baseline FS between 1 and 2, which is the most critical cutoff in SS histopathological evaluation.

The present study was prompted by a recent paper documenting that MSGB grading of inflammation was scarcely reproducible at different section depths, and that the difference between grades recorded at baseline and at deeper levels was sufficient to change the biopsy from positive to negative or vice versa in 10% of grade I (FS = 0), 44.4% of grade II (0 < FS < 1), 88.8% of grade III (1 ≤ FS < 2), and 40% of grade IV (FS ≥ 2) biopsies [13]. The authors of that paper recommended that multiple sections of MSGB should be examined to improve the reliability of the histopathological grading. However, they did not suggest how many sections should be examined or how to deal for diagnostic purposes with the different scores obtained at different levels, nor did they give a clinical interpretation of their results by entering them in a criteria set for SS patient classification.

On this basis, we aimed at assessing if the histopathological evaluation of a larger area of MSGB tissue, as obtained by cutting the biopsy sample at additional section levels, could increase the diagnostic performance of the histopathological study and of the AECG criteria set proposed for the classification of SS. We chose a minimum requirement of three different section levels, by analogy with the procedure standardized for the histopathological study of endomyocardial biopsies [15], assuming that a 200-μm distance should ensure the detection of independent foci on each section while reducing the chance of missing the smaller ones, thus allowing estimation of the overall density of inflammatory foci with sufficient precision.

With reference to the diagnostic gold standard, when patients were classified according to the AECG criteria set including the cFS, specificity increased by 9.8%, and the pairwise comparison of the ROC curves showed a statistically significant improvement of the diagnostic performance, mostly due to the increased test specificity in biopsies with 1 ≤ FS < 2, whereas the increase was minimal in FS ≥ 2 and null in biopsies inconsistent with SS (0 < FS < 1). One advantage of the proposed method of MSGB evaluation is that specificity is increased without affecting sensitivity; on the other hand, it was shown that improving sensitivity by means of increasing the cutoff value of positive FS resulted in a substantial reduction of specificity [16].

To explain the increased specificity observed with examination of multilevel salivary gland biopsies, it should be considered that, because of the uneven distribution of inflammatory infiltrates in the gland [14], the examination of a single tissue section might easily either overestimate or underestimate the FS, while the observation of a larger area of biopsy sample would allow a more precise quantification of the focus distribution, provided that the sections are distant enough to avoid recutting and rescoring of the same focus. In accordance with this hypothesis, and confirming previous results [13], after multilevel examination the higher numbers of FS changes proven to be relevant for classification and clinical diagnosis were seen in patients with mild to moderate MSGB inflammatory infiltrates (1 ≤ FS < 2), while very few relevant changes were recorded in patients with negative or highly positive biopsies (FS < 1 or FS ≥ 2). We suggest that in mild inflammation, lymphocytic foci are unevenly distributed through the gland, so that positive baseline sections can occasionally be followed by sections with less or no inflammation, whereas negative or highly positive biopsies (FS < 1 and ≥ 2) are likely to be more homogeneous. Our observations also confirmed the common knowledge that no single test can be reliably applied to the diagnosis of SS [2-9]. In fact, the performance of the test was significantly improved when the cFS was entered in the criteria set, but not when the histopathological test was considered alone.

One potential limit of the present study is represented by the need to introduce a gold standard reference to assess the diagnostic accuracy of the test, independent of the widely accepted AECG criteria set for SS classification. In fact, after clinical re-evaluation, which we adopted as a gold standard, some patients appeared to have been misclassified according to AECG criteria. This only partial correspondence between the judgement of experienced clinicians and classification criteria is a well-known problem in the diagnosis of rheumatological disorders and justifies the requirement of a wide criteria set for patient classification. In the absence of single, straightforward diagnostic parameters, a thorough patient's chart and follow-up revision by experienced rheumatologists was chosen as reference gold standard, by analogy with what has been done in many rheumatological studies, including that of the European Community Study Group on Diagnostic Criteria for SS [3-5]. Accordingly, a multicenter study would be useful to better standardize the procedure of evaluating FSs by oral pathologists, backed by a larger panel of experienced clinicians, because the clinical performance of SS classification criteria could be improved.

## Conclusion

The assessment of a cumulative focus score (cFS) obtained at three different section levels on minor salivary gland biopsies, cut at least 200 μm apart, can improve the diagnostic accuracy of the criteria set used for SS classification, especially in biopsies with a baseline FS between 1 and 2. Since the value of the MSGB biopsy has been confirmed by the recent AECG revision of the SS classification criteria [5], the increase of the diagnostic performance of the histological study will further help to correctly identify SS patients.

## Abbreviations

AECG = American-European Consensus Group; cFS = cumulative FS; CI = confidence interval; FS = focus score; MSGB = minor salivary gland biopsy; ROC = receiver operating characteristic; SE = standard error; SS = Sjögren's syndrome.

## Competing interests

The author(s) declare that they have no competing interests.

## Authors' contributions

PM participated in the design of the study, performed the histopathological analysis, coordinated the study, and drafted the manuscript. AM and RC reviewed and discussed patients' charts for clinical re-evaluation. OE performed all salivary gland biopsies. CV participated in case collection and data analysis. CT participated in the design of the study and performed the statistical analysis. ES and CM conceived the study and participated in its design. CM also participated in the clinical re-evaluation of patients. All authors read and approved the final manuscript.

## Figures and Tables

**Table 1:** Demographic, biopsy, and clinical data for 120 patients given salivary gland biopsies for Sjögren's syndrome (SS)

Clinical and laboratory parameters	FS^a ^= 0	0 < FS < 1	1 ≤ FS < 2	FS ≥ 2
No. of patients	57	11	15	37
Sex	9M/48F	1 M/10 F	0 M/15 F	3 M/34 F
Age (years)	46 ± 12	46 ± 11	54 ± 14	56 ± 13
Baseline biopsy area (mm^2^)	6.1 ± 5.6	8.3 ± 2.8	8.6 ± 4.3	4.7 ± 2.6
Cumulative area of three biopsies (mm^2^)	17.1 ± 15.1	21.8 ± 9.3	22.6 ± 12.7	12.9 ± 6.4
Findings [No. (%)]				
Dry eyes	49 (86)	9 (81)	14 (93)	33 (89)
Xerostomia	45 (79)	7 (63)	14 (93)	35 (94)
Positive Schirmer's test	24 (42)	4 (36)	9 (60)	18 (51)
Reduced salivary flow rate	30 (53)	7 (63)	13 (86)	34 (91)
Antinuclear antibodies	29 (51)	8 (73)	8 (53)	31 (83)
Ro/SS-A	18 (31)	3 (27)	6 (40)	25 (67)
La/SS-B	3 (5)	0	3 (20)	10 (27)
Rheumatoid factor	27 (47)	5 (45)	7 (46)	33 (89)
SS according to AECG criteria [No. (%)]	7 (12)	1 (9)	15 (100)	34 (92)
SS according to clinical re-evaluation [No. (%)]	7 (12)	0 (0)	8 (53)	34 (92)

^a^The focus score (FS) is the number of inflammatory infiltrates of at least 50 cells present in 4 mm^2 ^of salivary gland area. AECG, American-European Consensus Group; F, female; M, male; SS-A, anti-Ro60 antibodies; SS-B, anti-La antibodies.

**Table 2:** Changes in classification determined by multilevel salivary gland biopsies for Sjögren's syndrome (SS)

Test results and diagnostic accuracy	FS^a ^= 0	0 < FS < 1	1 ≤ FS < 2	FS ≥ 2	Total
No. of patients	57	11	15	37	120
AECG classification changes	0	0	6 (40°%)	1 (3%)	7
Baseline sensitivity (95% CI)	85.7% (42.2–97.6)	-	100% (62.9–100)	94.1% (80.3–99.1)	93.9% (83.1–98.6)
Revised sensitivity (95% CI)	85.7% (42.2–97.6)	-	100% (62.9–100)	94.1% (80.3–99.1)	93.9% (83.1–98.6)
Baseline specificity (95% CI)	98% (89.3–99.7)	-	0%^b^ (0–41.1)	33.3%^b^ (5.5–88.4)	84.5% (74.0–92.0)
Revised specificity (95% CI)	98% (89.3–99.7)	-	85.7% (42.2–97.6)	66.7% (11.6–94.5)	94.4% (86.2–98.4)

^a^The focus score (FS) is the number of inflammatory infiltrates of at least 50 cells present in 4 mm^2 ^of salivary gland area. ^b^Very low specificity is due to the absence (1 ≤ FS < 2) or extremely low number (FS ≥ 2) of patients classified as non-SS according to AECG criteria. AECG: American-European Consensus Group; CI: confidence interval; -, could not be evaluated with the available data.
